# Plasma phenylalanine and glutamine concentrations correlate with subsequent hepatocellular carcinoma occurrence in liver cirrhosis patients: an exploratory study

**DOI:** 10.1038/s41598-020-67971-x

**Published:** 2020-07-02

**Authors:** Kung-Hao Liang, Mei-Ling Cheng, Chi-Jen Lo, Yang-Hsiang Lin, Ming-Wei Lai, Wey-Ran Lin, Chau-Ting Yeh

**Affiliations:** 1 Department of Medical Research, Taipei Veterans General Hospital, Taipei, Taiwan; 2 Institute of Food Safety and Health Risk Assessment, National Yang-Ming University, Taipei, Taiwan; 3 Institute of Biomedical Informatics, National Yang-Ming University, Taipei, Taiwan; 4 Metabolomics Core Laboratory, Healthy Aging Research Center, Chang Gung University, Taoyuan City, 333 Taiwan; 5 Clinical Metabolomics Core Laboratory, Chang Gung Memorial Hospital, Taoyuan City, 33302 Taiwan; 6 Department of Biomedical Sciences, College of Medicine, Chang Gung University, Taoyuan City, 33302 Taiwan; 7 Liver Research Center, Chang Gung Memorial Hospital, Linkou, Taiwan; 8 Molecular Medicine Research Center, Chang Gung University, 5, Fu-Shin Street, Kuei-Shan District, Taoyuan, Taiwan

**Keywords:** Metabolomics, Liver

## Abstract

Aberrant metabolisms have been hypothesized to precede the occurrence of hepatocellular carcinoma (HCC), therefore, we investigated biomarkers associated with subsequent HCC in peripheral bloods using metabolomic technologies. A cohort of 475 HCC-naïve liver cirrhotic patients were recruited and prospectively followed. A total of 39 patients developed HCC in the follow-up period. Baseline plasma metabolites were explored using untargeted nuclear magnetic resonance. Candidates were then quantified by ultra-performance liquid chromatography. A series of univairiate and multivariate analysis showed that Phenylalanine (Phe) and Glutamine (Gln) levels are associated with time to HCC, independent of viological etiologies and age. A HCC risk score R was then constructed using the polynomial combination of age, Phe and Gln in the units of micromolar (μM):

$$\begin{aligned} {\text{R }} & = {\text{ Age }}* \, \left( {0.0694} \right) + {\text{ Phe }}* \, \left( {0.3399} \right) + {\text{ Phe }^{2}}* \, \left( { - 0.00188154} \right) \hfill \\ & \quad + {\text{ Gln }}* \, \left( { - 0.0133} \right) + {\text{ Gln }^{2}}* \, \left( { \, 0.00002244} \right) \hfill \\ \end{aligned}$$

R correlates with the time to HCC significantly (Hazard ratio [HR] = 2.368, 95% confidence interval [CI] 1.760–3.187, P < 0.001). An additional cross-sectional analysis showed that Phe and Gln concentrations both correlates with HCC occurrence in the next 3 years (area under the receiver operating characteristic curve [AUC] = 0.607 and 0.629, P = 0.033 and 0.010 respectively). In conclusion, phenylalanine and glutamine concentrations in the peripheral blood correlate with subsequent HCC.

## Introduction

Liver cirrhosis is a pathologic condition after decades of chronic hepatic necroinflammation, caused by either viral infections or chronic alcoholism. Once the liver become cirrhotic, the incidence of hepatocellular carcinoma (HCC) escalates significantly^1^. However, it seems unpredictable whether a cirrhosis person will develop HCC shortly after cirrhosis has commenced or will not develop HCC in their lifetime. The lack of patient stratification forces all cirrhosis patients to indiscriminately receive regular ultrasonography surveillance^2^. Unfortunately, the compliance is low among cirrhosis patients due to the insufficient awareness of the risk. HCC at early stages are often asymptomatic, consequently, many HCCs are diagnosed at intermediate or late stages.

As such, there is an unmet medical need to estimate the risk of HCC in liver cirrhosis patients. Metabolic disorders such as type 2 diabetes is one major etiology of HCC^3,4^. Even in the presence of viral infections, diabetes still independently raises the incidence of HCC^5^. Apart from diabetes, other aberrant metabolic processes may also precede the occurrence of HCC. The deregulated metabolites might be observed in the peripheral blood. Here we conducted a comprehensive exploratory screening of peripheral blood metabolites using the nuclear magnetic resonance technology (NMR). The concentrations of candidate metabolites were then quantified subsequently using ultra-performance liquid chromatography (UPLC).

## Results

### A HCC risk score was constructed using metabolite concentrations

Patient characteristics of this cohort was summarized in Table 1, including age, gender, etiology (hepatitis B virus [HBV] or hepatitis C virus [HCV] infection), aspartate transaminase (AST), alanine transaminase (ALT), AST/ALT ratio, platelet counts and fibrosis-4 (FIB-4) index^6^. A total of 39 patients developed HCC and diagnosed at the Barcelona Clinic Liver Cancer (BCLC) stage A.

**Table 1:** Demographic information of the study cohort.

	The cohort
Patient number	475
HCC (%)	39
Age (years)	59.50 ± 11.04
Gender—male (%)	298 (63%)
HBV (%)	273 (57%)
HCV (%)	149 (31%)
AST	44.50 ± 38.57
ALT	39.19 ± 43.27
AST/ALT	1.26 ± 0.48
Platelet	130.87 ± 57.54
FIB-4 index	4.51 ± 4.60

HBV, hepatitis B virus; HCV, hepatitis C virus; AST, aspartate transaminase; ALT, alanine transaminase; FIB-4, fibrosis-4.

**Figure 1:**
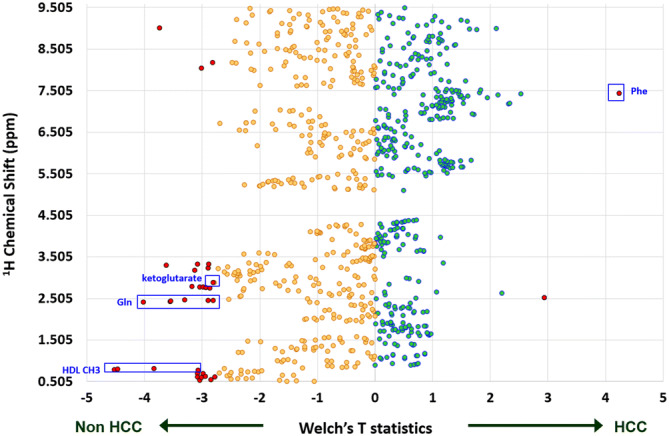
Metabolomic profiling of the cohort of liver cirrhosis patients. The vertical axis indicated the ^1^H nuclear magnetic resonance chemical shifts (between 0.505 and 9.495 ppm). The horizontal axis indicated Welsh’s t-statistics in the comparison of patients with or without the occurrence of HCC during follow-ups. Cyan and yellow dots represent positive or negative associations with HCC. Those with significant association (P ≤ 0.01) were particularly highlighted as red dots.

An untargeted metabolomic profiling was conducted using NMR on a wide spectrum of ^1^H chemical shifts (Fig. 1). Among the baseline clinical factors, age, HCV and AST were found to be associated with HCC occurrence in the univariate Cox-regression analysis (Table 2). Additionally, four metabolites was successfully annotated as phenylalanine (Phe), glutamine (Gln), high-density lipoprotein (HDL)-CH3, and ketoglutarate, based on the ^1^H chemical shifts significantly associated with HCC occurrence (P ≤ 0.01, Fig. 1). Phe levels were positively associated with HCC, while Gln, HDL-CH3 and ketoglutarate were negatively associated (Fig. 1). A multivariate Cox regression analysis (of the factors with P < 0.05 in the univariate analysis) showed that age, HCV, Phe and Gln were the factors independently associated with time-to-HCC (adjusted P < 0.001, = 0.030, 0.037 and 0.046 respectively, Table 2). Analyzing the 4 factors jointly, that Phe, Gln remained significantly associated with time-to-HCC (adjusted P = 0.037 and 0.011 respectively, Table 2), independent of age and HCV. Hence, Phe and Gln represent the metabolite candidates of this study.

**Table 2:** Clinical variables and metabolites in association with the time to HCC occurrence.

Variables	Univariate analysis				Multivariate analysis (7 variables)				Multivariate analysis (4 variables)			
	HR	95% CI		P	HR	95% CI		P	HR	95% CI		P
Age (years)	1.072	1.040	1.106	** < 0.001**	1.065	1.031	1.101	** < 0.001**	1.064	1.030	1.098	** < 0.001**
Gender—male	0.677	0.361	1.271	0.225								
HBV	0.698	0.372	1.308	0.261								
HCV	3.277	1.731	6.204	** < 0.001**	2.685	1.406	5.129	**0.030**	2.619	1.377	4.979	**0.030**
AST	1.004	1.001	1.008	**0.022**	1.002	0.997	1.008	0.366				
ALT	1.003	1.000	1.007	0.091								
AST/ALT	1.073	0.579	1.988	0.823								
Platelet	0.998	0.992	1.003	0.443								
FIB-4 index	1.039	0.996	1.085	0.079								
**Metabolites assessed by NMR**												
Phe	1.056	1.015	1.098	**0.006**	1.060	1.004	1.120	**0.037**	1.061	1.003	1.122	**0.037**
HDL-CH3	0.998	0.996	0.999	**0.005**	0.998	0.996	1.001	0.181				
Gln	0.984	0.973	0.995	**0.005**	0.986	0.972	1.000	**0.046**	0.986	0.975	0.997	**0.011**
Ketoglutarate	0.990	0.982	0.999	**0.022**	1.009	0.996	1.022	0.180				

P values less than 0.05 are indicated in bold.

HBV, hepatitis B virus; HCV, hepatitis C virus; AST, aspartate transaminase; ALT, alanine transaminase; FIB-4, fibrosis-4; Phe, phenylalanine; HDL, high-density lipoprotein; Gln, glutamine.

With the candidates being found in the NMR exploration, we further employed UPLC for the absolute quantification of the candidates using corresponding standards. The measurement from NMR and UPLC are highly correlated (Pearson’s correlation coefficients of Phe and Gln are 0.676 and 0.550 respectively, both P < 0.001, N = 475). A risk score R was derived using age and the Phe and Gln in the unit of micromolar (μM):

1 $$\begin{aligned} {\text{R}} & = {\text{age}}*\left( {0.0{694}} \right) + {\text{ Phe}}*\left( {0.{3399}} \right) + {\text{Phe}}^{{2}} *\left( { - 0.00{188154}} \right) \hfill \\ & \quad + {\text{Gln}}*\left( { - 0.0{133}} \right) + {\text{Gln}}^{{2}} *\left( {0.0000{2244}} \right) \hfill \\ \end{aligned}$$

The score reflect the hazards of the patient *i*:

2 $${\text{H}}\left( {{\text{t}}|{\text{R}}} \right) \, = {\text{ H}}_{0} \left( {\text{t}} \right){\text{e}}^{{({\text{R}})}}$$

**Figure 2:**
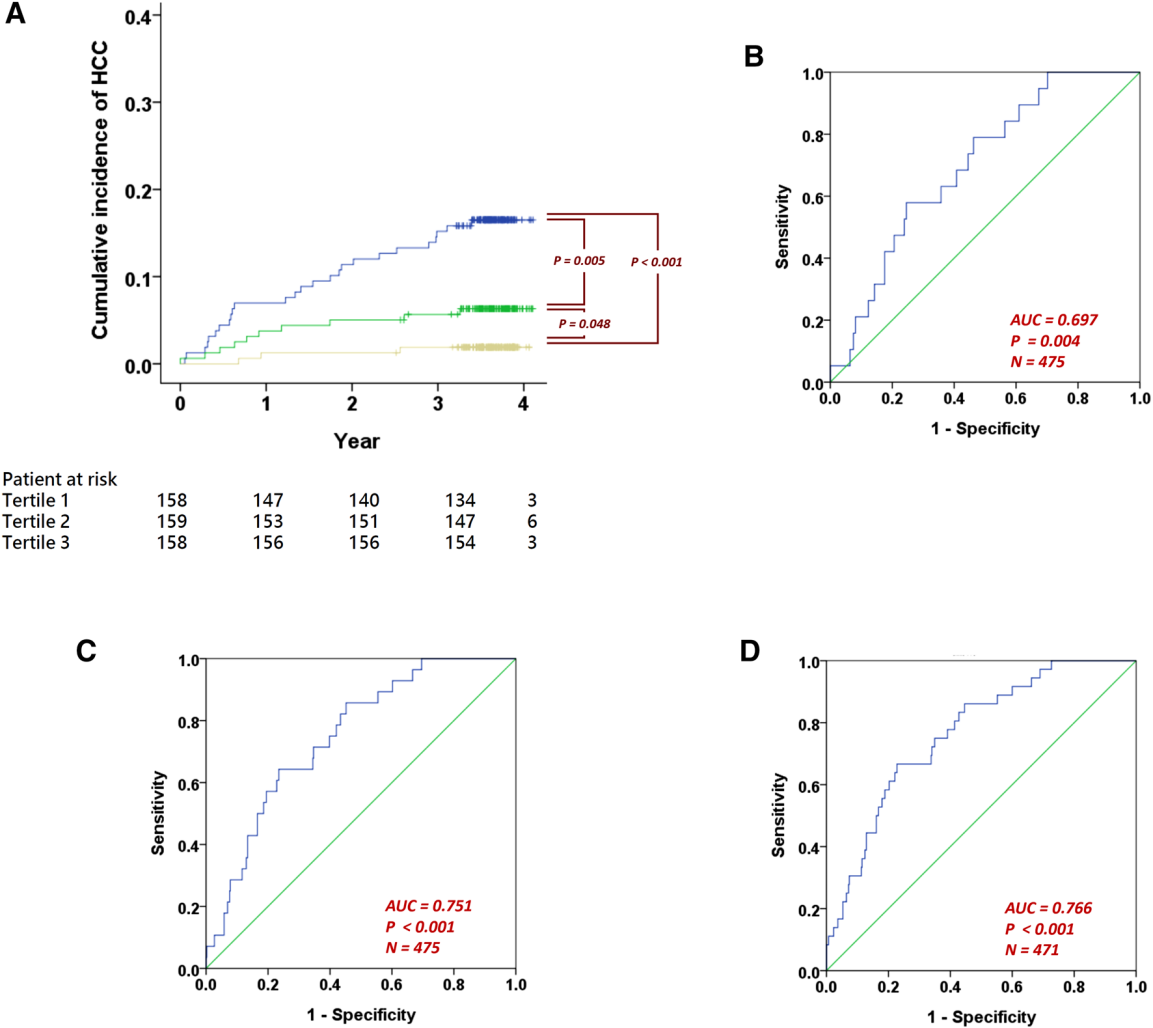
Risk score performance in time to event analysis and cross-sectional analysis. (**A**) The Kaplan–Meier plot of patients stratified into tertiles by the risk score (N = 475). (**B**) The receiver-operating-characteristic (ROC) curve of the risk score for classifying patients with or without HCC at 1 year after recruitment. The area under the ROC (AUC) is 0.697. (**C**) The ROC curve for classifying patients at 2 years after recruitment (AUC = 0.751). (**D**) The ROC curve for classifying patients at 3 years after recruitment (AUC = 0.766).

The risk score is significantly associated with time-to-HCC (HR = 2.368, CI 1.760–3.187, P < 0.001). Furthermore, distinct cumulative incidences of HCC were found in patient strata by the score (tertile 1 vs. tertile 3: P < 0.001; tertile 1 vs. tertile 2: P = 0.005; tertile 2 vs. tertile 3: P = 0.048; Fig. 2A).

### Correlations of the UPLC-quantified metabolites and the derived risk score with subsequent HCC occurrence at different time points

We then evaluated the performance of the risk score by a series of cross-sectional analysis using HCC status at years 1, 2 and 3 after baseline. The score can classify patients with or without HCC at 1 year successfully (area under the receiver-operating-characteristic curve [AUC] = 0.697, P = 0.004, Fig. 2B). The optimum cutoff which maximizes the Youden’s index^7^ is 18.543 (Table 3). At this cutoff, the sensitivity is 0.579 and the specificity is 0.754. The score can also be used for classifying patients’ status at 2 years (AUC = 0.751, P < 0.001, Fig. 2C). Interestingly, the same optimum cutoff was found (Table 3). The sensitivity is 0.643 and the specificity is 0.765. At year 3, the AUC is 0.766 (P < 0.001, Fig. 2D). The same cutoff was found again (Table 3). The sensitivity is 0.667 and the specificity is 0.772.

**Table 3:** The classification of HCC status at the subsequent years 1, 2 and 3 after patient recruitment.

	AUC	P	Performance at the cutoff which optimizes Youden's index					
			Cutoff	Youden's index	Sensitivity	Specificity	PPV	NPV
**Risk score**								
Year 1	0.697	0.004	18.543	0.333	0.579	0.754	0.089	0.977
Year 2	0.751	< 0.001	18.543	0.408	0.643	0.765	0.146	0.972
Year 3	0.766	< 0.001	18.543	0.439	0.667	0.772	0.195	0.966
**Phe**								
Year 1	0.580	NS						
Year 2	0.597	NS						
Year 3	0.607	0.033	66.320	0.252	0.806	0.446	0.107	0.965
**Gln**								
Year 1	0.638	0.041	39.398	0.307	0.737	0.570	0.067	0.981
Year 2	0.631	0.020	42.359	0.258	0.714	0.544	0.089	0.968
Year 3	0.629	0.010	39.398	0.239	0.667	0.572	0.114	0.954

**Figure 3:**
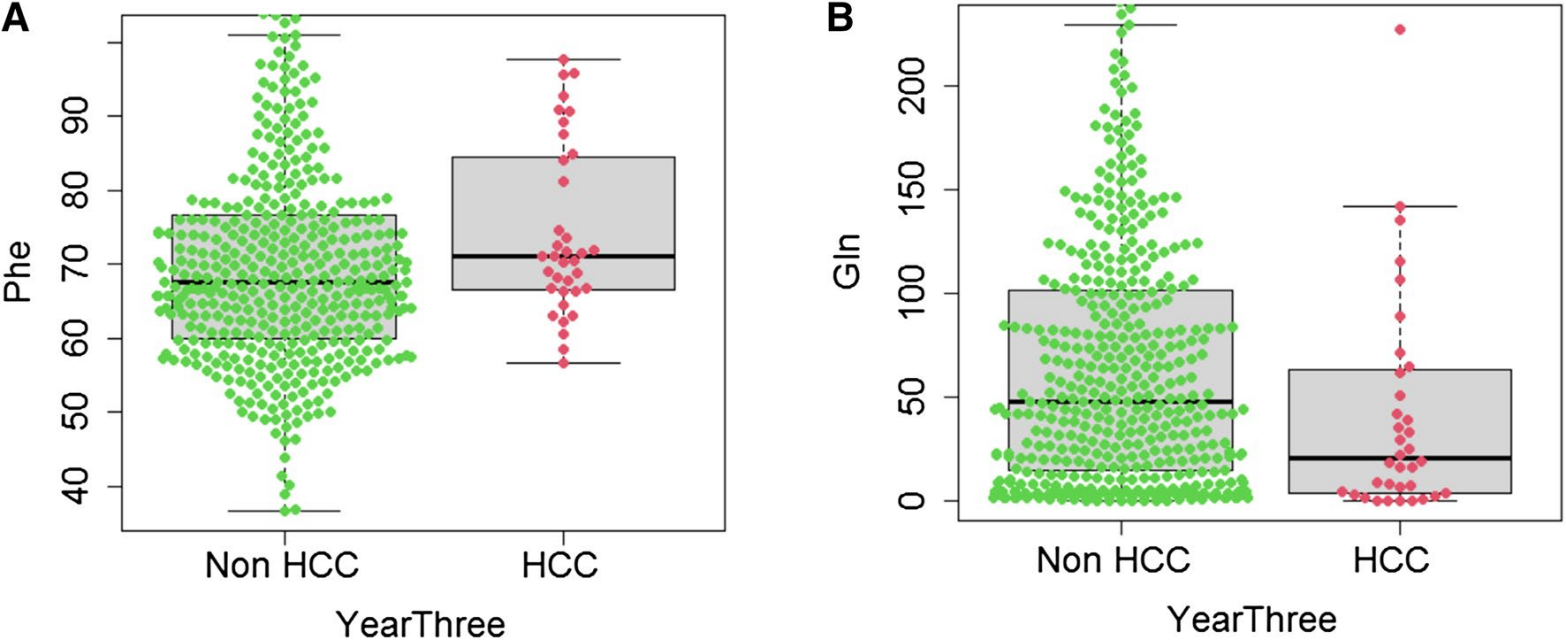
Box-and-Whisker plots overlaid with the Phe and Gln concentrations (μM) in patients who have or have not developed HCC at the third year. (**A**) The distribution of Phe concentrations (Mann–Whitney P = 0.033, non-HCC N = 435, HCC N = 36, censored N = 4). (**B**) The distribution of Gln concentrations (Mann–Whitney P = 0.010).

The Phe and Gln concentrations were also evaluated individually in terms of their performance in estimating patients’ status at subsequent time points. The Phe concentration is only significantly associated with the HCC status at year 3 (P = 0.033, AUC = 0.607, Table 3) but not at years 1 and 2 (AUC = 0.580 and 0.597 respectively). On the other hand, the Gln concentration is significantly associated with HCC at years 1, 2 and 3 (P = 0.041, 0.020 and 0.010 respectively, AUC = 0.638, 0.631 and 0.629 respectively, Table 3). The value distributions of Phe and Gln concentrations in patients with or without HCC at year three were visualized using Box-and-Whisker plots (Fig. 3).

## Discussion

Liver cirrhosis often precedes HCC, justifying the need for intensive ultrasound surveillance for all cirrhosis patients, a task difficult to achieve in many countries. Effective risk stratification in cirrhosis patients could lessen this medical burden. The NMR and ultra-performance liquid chromatography are two distinct platforms commonly used for exploring and quantifying metabolites. We used untargeted NMR for finding candidates associated with HCC. The candidates were then quantified using ultra-performance liquid chromatography with standards. Our NMR and UPLC measurements are highly correlated with each other. This approach led toward the discovery of Phe and Gln, the concentrations of which are associated with subsequent HCC occurrence in liver cirrhosis patients, independently of virological etiologies (Table 2). The relationship between these two amino acids and HCC has been sporadically reported in literature, mainly in cross-sectional studies rather than time-to-event analysis. Phe in the peripheral blood was elevated in HCC patients compared with that in liver cirrhosis patients^8^. Phe is an essential amino acid that can be metabolized into tyrosine by the phenylalanine hydroxylase (PAH). Abnormal metabolism of tyrosine, known as tyrosinemia, was reported to be linked to HCC occurrence^9^. Aberrant Gln metabolism has been implicated in metabolic syndrome, mitochondrial diseases as well as multiple cancers including HCC^10,11^. Gln can be metabolized to glutamate by glutaminases, GLS1 and GLS2, which have also been implicated to HCC^12^.

A risk score was constructed using age and the UPLC quantified values of Phe and Gln in a quadratic equation (Eq. 1). The quadratic equation was determined based on the time-to-event data using a computer-assisted, automatic GIM algorithm, which is capable of picking up any polynomial combination of variables, in an attempt to maximize the likelihood function as in the Cox-regression model. The risk score is significantly associated with time-to-HCC (HR = 2.368, CI 1.760–3.187, P < 0.001). We then performed a series of cross-sectional analysis to demonstrate that the risk score can classify patients with or without HCC at 1, 2 and 3 years from baseline (all P ≤ 0.004, Table 3).

We also evaluated the classification performance of Phe and Gln individually at these time points. Gln can successfully classify the HCC status at years 1, 2 and 3 from baseline (P = 0.041, 0.020 and 0.010 respectively, Table 3), while Phe can only classify patients at year 3 successfully (P = 0.033, Table 3). We took a closer look at the performance and found that balanced pairs of sensitivity and specificity were achieved consistently by Phe, Gln and the risk score. In contrast, high negative predictive values (NPV) and low positive predictive values (PPV) were achieved by the two biomarkers and the risk score (Table 3). The low PPV is caused by high false positives, which are the high-risk patients who have not developed HCC at the time point. This implied that the metabolite dis-regulation may have a slow, accumulating effect in HCC occurrence, with a time frame longer than we previously anticipated. Hence, this study is still limited by the insufficient duration of observation and also the insufficient sample size. It would also be interesting to examine the metabolite levels 3 month, 6 month or even night month before the diagnosis of HCC, provided that the HCC case numbers are large enough for showing the metabolite value distributions. This again requires longer observations in larger sample size, and remain to be our future research. Finally, this study is only an exploratory study where a validation study with an independent patient cohort is required in the future to confirm the findings.

In conclusion, age, Phe and Gln concentrations in plasma samples altogether offer a risk score which correlates with subsequent HCC occurrence in liver cirrhosis patients. Further validations on a larger cohort with longer duration of follow up is warranted.

## Methods

### Patients

This study was approved by the institutional review board of Chang Gung Memorial Hospital, Taiwan and conducted according to the principles in the declaration of Helsinki. A cohort of 475 consecutive HCC-naïve liver cirrhosis patients were recruited from three medical centers, the Keelung, Linko and Kaohsiung branches of Chang Gung Memorial Hospitals, which were located respectively in the northern, central-north and southern regions of Taiwan. The cirrhosis was diagnosed by either liver biopsy, or ultrasound imaging in conjunction with the detection of esophageal varices using endoscopy, or the transient elastography (Fibroscan; Echosens, France) measurements greater than 12 kilopascal (kPa). All patients were above 18 years old and have given informed consent. Peripheral bloods for the metabolomics study were collected between January, 2013 and August, 2014. These patients were then regularly followed in outpatient clinics every 3 months. Ultrasound survey were performed regularly until HCC was diagnosed. The end of follow-up is 2017/2/28. During the study period, all HBV patients have achieved sustained virological response. Patients with HCV were viremic.

Baseline clinical information such as age, gender, etiology (HBV, HCV), and aspartate Aminotransferase (AST), alanine Aminotransferase (ALT), AST/ALT and the Fibrosis 4 score (FIB-4)^6^ were retrieved from chart records. Patients were prospectively followed until the occurrence of HCC or the end of follow-up. A total of 39 patients developed HCC during the follow up time. The peripheral blood samples were centrifuged for the separation of plasma, which were then stored in − 20 °C until further analysis.

### Untargeted metabolomics using NMR

The plasma sample (350 μl) was mixed with 350 μl of plasma buffer solution (75 mM Na_2_HPO_4_, 0.08% TSP, 2 mM NaN_3_, 20% D_2_O), and 600 μl of the supernatant was transferred to NMR tubes for data acquisition.

^1^H NMR spectra were acquired on a Bruker Avance III HD 600 MHz NMR spectrometer with a 5 mm inverse triple resonance CryoProbe (^1^H/^13^C/^15^N) (Bruker Biospin GmbH, Rheinstetten, Germany). The spectra were acquired by Carr-Purcell-Meiboom-Gill spin-echo (CPMG) pulse sequence at 310 K, and broad signals from proteins were attenuated by the 80 ms T_2_ relaxation time. The spectrum was collected with a spectral width of 12,019.23 Hz and 72 k data points and then acquisitions were accumulated 64 times. All NMR spectra were phased and baseline-corrected and then referenced to the doublet of ^1^H-α-glucose at 5.23 ppm by using Topspin software (version 3.2.2; Bruker Biospin GmbH, Rheinstetten, Germany)^13^. We chose CPMG pulse as a compromise of efficiency and effectiveness for the current study. The utilization of NOESY, PURGE, PROJECT pulses and other technique remain our future research^14^.

Each ^1^H NMR spectrum (in the range of 9.5–0.5 ppm, excluding the water region) from plasma was segmented into 0.01 ppm with equal widths, and normalized to the reference by AMIX (version 3.9.14; Bruker Biospin GmbH, Rheinstetten, Germany). The resulting data sets were analyzed by SIMCA-P+ (version 13.0; Umetrics, Umea, Sweden), and all data were Pareto-scaled for multivariate statistical analysis. Resonant frequencies of each metabolite were referred from an in-house library, Chenomx NMR Sutie 7.1 (Chenomx, Edomonton, Canada), or HMDB (https://www.hmdb.ca/)^15^. More technical details can be found in literature^13^.

### Ultra-performance liquid chromatography (UPLC)-based amino acid measurement

The plasma samples (100 µl) were precipitated by adding an equal volume (100 µl) of 10% sulfosalicylic acid containing an internal standard (norvaline 200 µM)^16^. The 20 µl of the supernatant was mixed with 60 µl borate buffer (pH 8.8) and then the derivatization was activated by adding 20 µl of 10 mM AQC in acetonitrile. After 10 min reaction time, the reaction was disrupted by mixing with an equal volume of Eluent A (20 mM ammonium formate/0.6% Formic acid/1% acetonitrile) and analyzed using the ACQUITY UPLC System. The AQC derivatization reagent was obtained from the Waters Corporation (Milford, MA, USA)^17^.

The Waters ACQUITY UPLC System (Waters crop., Milford, USA) consisted of a Binary Solvent Manager (BSM), a Sample Manager fitted with a 10-µl loop, and a Tunable UV (TUV) detector. The system was controlled, and the data was collected using Empower 2 software. The separations were performed on a 2.1 × 100 mm ACQUITY BEH C18 column at 60 °C and flow rate of 0.70 ml/min, and the detection was set at 260 nm using a sampling rate of 20 points/s. The mobile phase was 20 mM ammonium formate/0.6% formic acid/1% acetonitrile in water (Eluent A) and in acetonitrile (Eluent B)^15^.

### Data visualization and analysis

Clinical variables were compared using Welch’s t test (*a.k.a.* unequal variances t-statistics), Mann–Whitney U test and χ^2^ test, where the obtained P values smaller than 0.05 were considered statistical significance. The result of NMR exploration was presented using a scatter plot of the Welch’s t statistics and the ^1^H chemical shifts. Welch’s t-test were performed on patients with or without the occurrence of HCC during the follow-up. Cox regressions were used for univariate and multivariate analysis of clinical and metabolic variables for their correlation with the time to HCC. Cumulative incidence of HCC of different patient strata were compared using log-rank tests. The IBM SPSS software version 20 (IBM, Armonk, NY) was used. The Box-and-Whisker plots were produced by the R statistical package. The HCC risk models were constructed by the multivariate combination of variables using the generalized iterative modelling method (GIM). This algorithm can identify optimum polynomial combinations of variables with respect to the fitness function^18,19^, which in this research is the likelihood function as in the Cox regression. The software code of GIM (capable of doing the time-to-event analysis) can be downloaded at the following website (https://github.com/khliang/GIM).

## Abbreviations

HCC: Hepatocellular carcinoma
NMR: Nuclear magnetic resonance
UPLC: Ultra-performance liquid chromatography
HBV: Hepatitis B virus
HCV: Hepatitis C virus
AST: Aspartate transaminase
ALT: Alanine transaminase
FIB-4: Fibrosis-4
Phe: Phenylalanine
HDL: High-density lipoprotein
Gln: Glutamine
